# Disclosing HIV status to sexual partner: Findings from a People Living with HIV Stigma Index 2.0 study in the country Georgia

**DOI:** 10.1371/journal.pone.0331919

**Published:** 2025-10-08

**Authors:** Tamar Zurashvili, Mariam Pashalishvili, Valerie A. Earnshaw, Hyungrok Do, Natalia Zakareishvili, Jack DeHovitz, Suzan M. Walters, Mamuka Djibuti

**Affiliations:** 1 Faculty of Medicine, Ivane Javakhishvili Tbilisi State University, Tbilisi, Georgia; 2 Partnership for Research and Action for Health, Tbilisi, Georgia; 3 Department of Human Development and Family Sciences, University of Delaware, Newark, Delaware, United States of America; 4 New York University Grossman School of Medicine, New York, New York, United States of America; 5 UNFPA Georgia, United Nations Population Fund, Tbilisi, Georgia; 6 Department of Medicine, SUNY Downstate Health Sciences University, Brooklyn, New York, United States of America; Virginia Mason Franciscan Health, UNITED STATES OF AMERICA

## Abstract

**Background:**

HIV status disclosure to sexual partner plays an important role in fostering transparency and reducing stigma, yet it remains a complex issue influenced by various sociodemographic, psychosocial, and experiential factors. This study investigated factors associated with HIV status disclosure to sexual partner among people living with HIV (PLHIV) in Georgia.

**Methods:**

We conducted a secondary analysis of data from the PLHIV Stigma Index 2.0 study conducted in 2022–2023. Participants were recruited from HIV care centers and community-based organizations. Data collection utilized standardized questionnaires assessing sociodemographic factors, stigma, discrimination, and interactions with the healthcare system. Statistical analysis employed descriptive statistics, bivariate, and multivariate logistic regression to examine associations between stigma, sociodemographic factors, and status disclosure to sexual partner.

**Results:**

Out of 765 participants, the mean age was 40.6 years, with a majority being male (67.4%). More than a fifth of respondents reported treatment interruptions, with 35.3% not disclosing their status to sexual partner. Disclosure was more common to close contacts than to others. Indicators of internalized stigma were common, with participants reporting feelings of guilt (40.1%), shame (36.1%), worthlessness (28.4%), and feeling ‘dirty’ (12.4%). Common behavioral reactions to stigma included avoiding medical visits (13.1%) and refraining from social support (10.5%). Over 40% did not disclose their status to sexual partner. Logistic regression highlighted that older age, knowing partner’s HIV status, positive disclosure experiences and enacted stigma were positively associated with status disclosure.

**Conclusion:**

The complex dynamics between stigma and HIV status disclosure highlight the importance of providing decision support to PLHIV, helping them navigate disclosure process while considering potential risks and benefits. The findings emphasize the need for focused interventions that promote disclosure to sexual partner, especially among individuals with treatment interruptions, as it can significantly impact personal health and broader public health objectives, including the prevention of HIV transmission.

## Background

HIV/AIDS poses a significant global and regional challenge, with Eastern Europe and Central Asia (EECA) experiencing the world’s fastest-growing epidemic [1]. Despite a 39% global decrease in new HIV infections since 2010, EECA has witnessed a 20% surge during the same period, paralleled by a 34% rise in AIDS-related deaths, contrasting with the global trend of a 51% reduction since 2010 [1]. The HIV epidemic in EECA is exacerbated by pervasive stigma and discrimination, which serve as social determinants that drive health inequities [2], create barriers to accessing essential HIV prevention and treatment services, and hinder the region’s ability to effectively address the HIV crisis [1,3,4].

Stigma is a driver of HIV inequities and a social and structural determinant of health [5]. Aimed to reduce the global HIV burden, the Global AIDS Strategy 2021–2026 has set a goal for countries to implement reforms to reduce stigma to fewer than 10% of PLHIV and other key populations (KPs) experiencing stigma and discrimination by 2025. However, the PLHIV Stigma Index 2.0 global report indicates that a substantial 84.8% of respondents sampled between 2020 and 2023 in 25 countries reported internalized stigma due to HIV, suggesting that the 2025 goal is likely to be unmet [6].

HIV stigma is a social process supported by power that results in social devaluation of PLHIV [7]. Discrimination is a manifestation of this social process that includes unjust or unfair behaviors directed towards PLHIV [8]. Stigma and discrimination ultimately lead to adverse psychological, behavioral, and health outcomes among PLHIV. Despite decades of scientific advances in prevention and treatment, as well as widespread awareness-raising efforts, irrational fears of HIV infection and negative attitudes towards PLHIV are a persistent barrier to addressing the epidemic and undermine interventions across the HIV prevention, treatment and care continuum [9]. As a result of stigma and discrimination, people often refuse to get tested for HIV, PLHIV have been denied access to HIV prevention and treatment services and stigma affects their ability to stay adherent to antiretroviral treatment (ART) [10–14]. This is reflected in the HIV care cascade data in the EECA region with only 59% [48–67%] of PLHIV knowing their status, 50% [41–57%] receiving ART, and 42% [39–46%] achieving viral suppression [1].

HIV disclosure, where individuals living with HIV reveal their status to others, comes with various challenges. It can negatively impact self-esteem and increase fear, making people reluctant to share their status [15]. However, the process also offers several benefits. It can provide relief, strengthen relationships, foster empowerment, and enable access to effective treatment and support [16]. Disclosure is a crucial public health goal because it can motivate sexual partners to seek testing and reduce HIV transmission, while also providing individuals with social support, improved medical care access, opportunities for risk reduction discussions with partners, and future planning [17].

### Georgia: Local context

Georgia remains a low HIV prevalence country with concentrated epidemics among KPs. Despite low HIV prevalence (0,4%) in the general population and signs of a decreasing trend of new HIV diagnoses, the evidence of high HIV levels in men who have sex with men (MSM) and transgender people, rates of recent HIV transmission in MSM and adolescents with high-risk behaviors indicate ongoing transmission and risk of potential worsening of the HIV epidemic [18]. Research on HIV-related stigma in Georgia is limited, with only a few qualitative studies conducted among youth, key populations, and healthcare providers in 2020 [19,20]. In 2022, the first comprehensive attempt to estimate stigma and discrimination among PLHIV in Georgia was undertaken through a large-scale PLHIV Stigma Index 2.0 study conducted by a PLHIV community-based organization.

Factors such as HIV/AIDS-related stigma, discrimination, and their impact on HIV status disclosure remains underexplored in Georgia. In order to further explore this we will draw upon Chaudoir and Fisher’s Disclosure Process Model [21] and utilize a globally recognized tool – PLHIV Stigma Index 2.0 [22] – to assess the complex interplay of stigma, discrimination and status disclosure.

## Methods

Using the PLHIV Stigma Index 2.0 we explored the relationship between stigma and HIV status disclosure to sexual partner. The cross-sectional study was carried out in Georgia in 2022–2023 by the community-based organization ‘Real People Real Vision’. The study used a standard methodology developed in collaboration with UNAIDS, ensuring consistency in data collection and analysis across different countries. This methodology involves a participatory approach, where PLHIV are trained to conduct peer-to-peer interviews with other individuals living with HIV. The Stigma Index measures multiple dimensions of stigma, including enacted and internalized stigma, behavioral reactions to stigma, experiences of discrimination in healthcare, employment, and social settings, and its impact on relationships, and access to services [23]. The data for this secondary analysis were accessed in November 2023.

### Participants

Participants were recruited from two primary sources: The Infectious Diseases, AIDS and Clinical Immunology Research Centers and non-governmental organizations (NGO) providing HIV prevention services to KPs in the capital city Tbilisi and in selected regions, including Adjara, Imereti and Samegrelo-Zemo Svaneti. Regions were selected based on the geographical distribution of PLHIV, ensuring representation from areas with the highest, mid-level, and lowest HIV prevalence, covering both east and west Georgia and accounting for 61% of the estimated number of PLHIV in the country. The planned sample size was 750 PLHIV, determined using the People Living with HIV Stigma Index 2.0 calculator [24], considering a target precision of 5% and a confidence level of 95%. Recruitment spanned regions with varying HIV prevalence, ensuring a proportional representation of registered PLHIV in each area. Venue-based sampling and Limited chain referral sampling approaches were employed for participant selection in line with the abovementioned standard methodology. Refusal rate was reported at 10%.

Criteria for inclusion included age 18 or above, mental competence to provide informed consent, and ability to speak and understand in the Georgian language. Exclusion criteria encompassed individuals under 18, language comprehension issues, physical or mental disabilities hindering participation, refusal to provide consent, and representation of KPs not living with HIV.

### Data collection

Data were collected from October 2022 to August 2023. Data collection employed the PLHIV Stigma Index 2.0 standard questionnaire, covering aspects such as demographics, stigma and discrimination experiences, interactions with healthcare system, human rights, and non-HIV-related discrimination. Interviews were conducted in suitable venues with trained interviewers, who were also PLHIV, ensuring privacy, comfort, and adherence to COVID-19 safety measures.

### Measures

The outcome measure for this study was HIV status disclosure to a sexual partner, assessed through a question asking whether husband, wife, or partner(s) knew the respondent’s HIV status. If the response was “Yes,” a follow-up question determined whether their status had ever been disclosed to this person or group without their consent. Cases where disclosure occurred without the respondent’s consent were excluded from the analysis, leaving only those who voluntarily disclosed their status. The rationale behind this decision was that unauthorized disclosure does not provide insight into the respondent’s willingness or decision-making regarding disclosure or non-disclosure. In these instances, the individual was not the agent of disclosure, making it unclear whether they would have disclosed or not voluntarily under different circumstances. Including such cases in the analysis could misrepresent the true patterns of voluntary disclosure and introduce bias in assessing the factors influencing disclosure decisions. Overall, 396 participants indicated that they voluntarily disclosed their HIV status to husband/wife/sexual partner(s), while 269 indicated that they have not; 43 participants with unauthorized disclosure where excluded from the analysis.

### Sociodemographic and HIV-related characteristics

The sociodemographic characteristics included participants’ age (categorized into four groups: 18–29, 30–39, 40–49, and 50 or older), sex at birth (male/female), which were derived from the original PLHIV Stigma Index. Grouping for education level (classified into two categories: Lower level of education [encompassing primary, secondary, vocational education and no formal education], and higher level of education [indicating university education]) and employment status (categorized into two groups: having some form of employment [comprising those in full-time or part-time work as an employee, self-employed or business owners working full-time, and those engaged in casual or informal part-time work] and unemployed [encompassing those who were unemployed or retired/on pension]), were created for this study. The original questionnaire asked participants about their belonging to certain minority groups (such as a racial, ethnic, or religious minority, people living with a disability, refugee or asylum seeker, migrant worker, internally displaced person, incarcerated/in prison) or support group of PLHIV. We also grouped the length of time participants knew their HIV status into four categories: less than 2 years, 2–5 years, 6–10 years, and more than 10 years. Additionally, we considered whether participants were currently in a sexual relationship (yes/no) and their partner’s HIV status (positive, negative, or not aware of partner’s HIV status), as reported in the original PLHIV Stigma Index.

### Experience with status disclosure

The measurement of participants’ experiences with HIV status disclosure encompassed five key statements from the original study, each with response options of ‘Agree,’ ‘Somewhat Agree,’ and ‘Disagree.’ Participants were asked: (1) whether disclosing their HIV status to people they were close to (e.g., partners, family, close friends) had been a positive experience, (2) if these close contacts were supportive upon learning about their HIV status, (3) whether disclosing their HIV status to people they did not know very well had been a positive experience, (4) if individuals with whom they had limited familiarity were supportive upon learning about their HIV status, and (5) whether the act of disclosing their HIV status had become progressively easier over time. To quantify experiences across these disclosure-related questions, we created composite scores for the disclosure experience domain. This domain consisted of the five variables mentioned, and responses were assigned numerical values: ‘Disagree’ was scored as −2, ‘Somewhat Agree’ was scored as 1, and ‘Agree’ was scored as 2 (Cronbach’s Alpha: 0.7933).

### Enacted stigma

We derived nine questions from the original study measuring enacted stigma. The questions included: (1) Have you ever been excluded from social gatherings or activities (e.g., weddings, funerals, parties, clubs) because of your HIV status? (2) Have you ever been excluded from religious activities or places of worship because of your HIV status? (3) Have you ever been excluded from family activities because of your HIV status? (4) Have you ever been aware of family members making discriminatory remarks or gossiping about you because of your HIV status? (5) Have you ever been aware of other people (other than family members) making discriminatory remarks or gossiping about you because of your HIV status? (6) Has someone ever verbally harassed you (e.g., yelled, scolded, or was otherwise verbally abusive) because of your HIV status? (7) Has someone ever physically harassed or hurt you (e.g., pushed, hit, or was otherwise physically abusive) because of your HIV status? (8) Have you ever been refused employment or lost a source of income or job because of your HIV status? (9) Has your wife/husband, partner(s), or child(ren) ever experienced discrimination because of your HIV status? Response options for all questions were: Yes, Within the last 12 months; Yes, but not in the last 12 months; and No. We created composite scores for the enacted stigma domain. This domain consisted of the nine variables mentioned, and responses were scored based on the timeliness and occurrence of such experiences: a response of ‘No’ was scored as −2, ‘Yes but not in the last 12 months’ was scored as 1, and ‘Yes in the last 12 months’ was scored as 2 (Cronbach’s Alpha: 0.7143).

### The impact of HIV status on various aspects of respondents’ lives

For the measurement of the impact of HIV status on respondents’ lives we used eight questions from the original study. Participants were asked to reflect on whether their HIV status had positively, negatively, or not affected various aspects of their lives, including: (1) My self-confidence; (2) My self-respect; (3) My ability to respect others; (4) My ability to cope with stress; (5) My ability to have close and secure relationships with others; (6) My ability to find love; (7) My desire to have children; and (8) My ability to achieve personal and/or professional goals. Response options for all questions included: Has been positively affected by my HIV status; Has not been affected by my HIV status; and has been negatively affected by my HIV status. We created composite scores for this study within the impact on lives domain. This domain consisted of the eight variables mentioned, with responses scored as follows: a response of ‘Negative’ was scored as −1, ‘No Impact’ as 0, and ‘Positive’ as 1 (Cronbach’s Alpha: 0.8272).

### Behavioral reactions to HIV stigma

The assessment of behavioral reactions to stigma based on the PLHIV stigma index consisted of six questions, focusing on participants’ actions over the past 12 months: (1) I have chosen not to attend social gatherings; (2) I avoided going to a clinic or hospital when I needed to; (3) I have chosen not to apply for a job(s); (4) I have chosen not to seek social support; (5) I have isolated myself from family and/or friends; and (6) I decided not to have sex. Responses were recorded as ‘Yes’ or ‘No,’ where positive answers indicated behavioral reactions to stigma. To quantify the extent of these actions, we created composite scores for this study. This domain comprised the six variables mentioned, with responses scored as follows: a response of ‘No’ was assigned a score of −1, while ‘Yes’ received a score of 1 (Cronbach’s Alpha: 0.7717).

### Internalized stigma

Internalized stigma, as defined by the PLHIV stigma index, was measured through a series of six statements designed to capture the extent to which participants internalized negative beliefs and emotions about their HIV status. Participants were asked to indicate their agreement with the following statements: (1) It is difficult to tell people that I am HIV positive; (2) Being HIV positive makes me feel dirty; (3) I feel guilty that I am HIV positive; (4) I am ashamed that I am HIV positive; (5) I sometimes feel worthless because I am HIV positive; and (6) I hide my HIV status from others. The response options were ‘Yes’ or ‘No’. To further analyze this domain, we created composite scores, quantifying the degree of internalized stigma based on participant responses. This domain consisted of six variables mentioned, and responses were scored with ‘No’ receiving a score of −1 and ‘Yes’ receiving a score of 1 (Cronbach’s Alpha: 0.7059).

### HIV testing and treatment experience

HIV testing and treatment experiences were captured through a series of questions asking whether their HIV testing was a personal choice or not (for example, being tested without being informed, being forced, or was born with HIV) and the main reason behind undergoing the test. Additionally, participants indicated if fears about potential reactions from family, friends, employers, or the community influenced their decision to get tested for HIV. Participants also reported on their current or past use of HIV treatment and the experiences with treatment interruptions.

### Ethical considerations

The original study received approval from the National Center for Disease Control and Public Health of Georgia IRB (IRB # 2022−082). Written informed consent was obtained from all participants, emphasizing voluntariness and confidentiality. Measures were in place to ensure confidentiality, including staff agreements, training, unique identifiers, and private interview settings. The study adhered to ethical standards, respecting participants’ rights, and ensuring no adverse impact on their services.

For the present secondary data analysis, additional ethical approval was obtained from the same IRB (IRB #2023-071). No research activities related to this analysis were undertaken until formal approval was granted.

### Statistical analysis

Descriptive statistics were employed to summarize sociodemographic characteristics, stigma and discrimination, and other factors, with frequencies and percentages calculated for each categorical variable. Composite scores were computed to assess overall stigma and discrimination levels. To compare the characteristics between individuals who disclose their HIV status to sexual partner and those who do not, chi-square test was employed for categorical variables, with Fisher’s exact test utilized for cells with fewer than five observations and independent samples t-test was used for the stigma composite scores. Associations between variables were assessed using unadjusted odds ratios (ORs), with the adjusted ORs (aORs) obtained from multivariable logistic regression model controlling for potential confounders. Variables were selected for inclusion in the model based on their statistical significance in the bivariate analysis and their relevance to the pre-specified hypotheses. The 95% confidence intervals (CI) for the ORs were calculated to provide a range of plausible values for the effect sizes. Statistical significance was determined using p-values. A p-value of less than 0.05 was considered statistically significant for all tests. For the assessment of reliability of composite scores for different stigma domains we calculated internal consistency reliability coefficient (Cronbach’s Alpha) for each domain. All statistical analyses were performed using R software [25].

## Results

### Main characteristics of study participants

Table 1 outlines the main characteristics of the study participants. Overall, 765 respondents completed the survey. The mean age of participants was 40.6 years (SD = 10.8), with most (n = 469; 61.3%) falling within the 30–49 age group, 67.4% (n = 509) were male and 65.4% (n = 497) had lower level of education. Employment status varied, with 302 (39.6%) participants being unemployed and 460 (60.4%) engaged in some form of employment. A majority of respondents (n = 470; 61.4%) reported being in an intimate or sexual relationship, out of which 218 (46.5%) indicated having an HIV-negative partner and 36 (7.7%) were unsure of their partners’ HIV status.

**Table 1:** Sample characteristics (N = 765).

Variable	n/N	%
**Sociodemographic**		
**Sex at birth** *(10 missing data)*		
Male	509/755	67.4
Female	246/755	32.6
**Age group**		
18-29	131/765	17.1
30-39	248/765	32.4
40-49	221/765	28.9
50+	165/765	21.6
**Length of time knowing HIV status** *(3 missing data)*		
Less than 2 years	37/762	4.9
2-5 years	276/762	36.2
6-10 years	229/762	30.0
More than 10 years	195/762	25.6
Do not remember	25/762	3.3
**Currently in an intimate/sexual relationship (whether married or unmarried)**		
No	295/765	38.6
Yes	470/765	61.4
**Partner’s HIV status** *(out of 470 who responded to the previous question, 1 missing data)*		
Negative	218/469	46.5
Positive	215/469	45.8
Unsure about the HIV status of partner	36/469	7.7
**Education level** *(5 missing data)*		
Lower level education	497/760	65.4
Higher level education	263/760	34.6
**Current work status** *(3 missing data)*		
Unemployed	302/762	39.6
Having some form of employment	460/762	60.4
**How often have you been unable to meet basic needs** *(8 missing data)*		
Never	160/757	21.1
Some of the time	463/757	61.2
Most of the time	134/757	17.7
**Disclosure experiences**		
**Had voluntarily disclosed status to husband/wife/sexual partner(s)** *(43 participants with unauthorized disclosure and 35 who indicated N/A were excluded, 22 missing data)*		
Yes	396/665	59.5
No	269/665	40.5
**Had voluntarily disclosed status to children** *(25 participants with unauthorized disclosure and 95 who indicated N/A were excluded, 41 missing data)*		
Yes	129/604	21.4
No	475/604	78.6
**Had voluntarily disclosed status to other family members** *(70 participants with unauthorized disclosure and 6 who indicated N/A were excluded, 25 missing data)*		
Yes	362/664	54.5
No	302/664	45.5
**Had voluntarily disclosed status to friends** *(62 participants with unauthorized disclosure and 6 who indicated N/A were excluded, 25 missing data)*		
Yes	257/668	38.5
No	411/668	61.5
**Had voluntarily disclosed status to neighbors** *(27 participants with unauthorized disclosure and 8 who indicated N/A were excluded, 45 missing data)*		
Yes	43/685	6.3
No	642/685	93.7
**Had voluntarily disclosed status to employer(s)** *(15 participants with unauthorized disclosure and 26 who indicated N/A were excluded, 50 missing data)*		
Yes	27/674	4.0
No	647/674	96.0
**Had voluntarily disclosed status to co-workers** *(14 participants with unauthorized disclosure and 30 who indicated N/A were excluded, 50 missing data)*		
Yes	34/671	5.1
No	637/671	94.9
**Had voluntarily disclosed status to teacher(s)/school administrator(s)** *(12 participants with unauthorized disclosure and 108 who indicated N/A were excluded, 54 missing data*		
Yes	6/591	1.0
No	585/591	99.0
**Had voluntarily disclosed status to classmates** *(11 participants with unauthorized disclosure and 107 who indicated N/A were excluded, 55 missing data)*		
Yes	7/592	1.2
No	585/592	98.8
**Had voluntarily disclosed status to local leaders** *(11 participants with unauthorized disclosure and 35 who indicated N/A were excluded, 53 missing data)*		
Yes	5/666	0.8
No	661/666	99.2
**Had voluntarily disclosed status to authority figures** *(13 participants with unauthorized disclosure and 27 who indicated N/A were excluded, 53 missing data)*		
Yes	15/672	2.2
No	657/672	97.8
**Disclosing your HIV status to people you are close to has been a positive experience** *(25 participant who indicated N/A [without such experience] were excluded, 1 missing data)*		
Disagree	149/739	20.2
Agree	325/739	44.0
Somewhat agree	265/739	35.8
**People you are close to were supportive when they first learned about your HIV status** *(32 participant who indicated N/A [without such experience] were excluded, 2 missing data)*		
Disagree	160/731	21.9
Agree	335/731	45.8
Somewhat agree	236/731	32.3
**Disclosing your HIV status to people you don’t know very well has been a positive experience** *(101 participant who indicated N/A [without such experience] were excluded, 3 missing data)*		
Disagree	397/661	60.1
Agree	76/661	11.5
Somewhat agree	190/661	28.7
**People you don’t know very well were supportive when they first learned about your HIV status** *(91 participant who indicated N/A [without such experience] were excluded, 1 missing data)*		
Disagree	371/673	55.1
Agree	105/673	15.6
Somewhat agree	197/673	29.3
**Disclosing your HIV status has become easier over time** *(22 participant who indicated N/A [disclosure was easy from the outset] were excluded, 2 missing data)*		
Disagree	416/741	56.1
Agree	137/741	18.5
Somewhat agree	188/741	25.4
**Enacted Stigma**		
**Have you ever been excluded from social gatherings or activities because of your HIV status?** *(41 participant who indicated N/A were excluded, 1 missing data)*		
No	701/723	97.0
Yes	22/723	3.0
**Have you ever been excluded from religious activities or places of worship because of your HIV status?** *(45 participant who indicated N/A were excluded, 1 missing data)*		
No	705/719	98.0
Yes	14/719	2.0
**Have you ever been excluded from family activities because of your HIV status?** *(41 participant who indicated N/A were excluded, 1 missing data)*		
No	696/723	96.3
Yes	27/723	3.7
**Have you ever been aware of family members making discriminatory remarks or gossiping about you because of your HIV status?** *(42 participant who indicated N/A were excluded, 1 missing data)*		
No	651/722	90.1
Yes	71/722	9.9
**Have you ever been aware of other people (other than family members) making discriminatory remarks or gossiping about you because of your HIV status?** *(37 participant who indicated N/A were excluded, 1 missing data)*		
No	625/727	86.0
Yes	102/727	14.0
**Has someone ever verbally harassed you (e.g., yelled, scolded, or was otherwise verbally abusive) because of your HIV status?** *(28 participant who indicated N/A were excluded, 1 missing data)*		
No	683/736	93.0
Yes	53/736	7.0
**Has someone every physically harassed or hurt you (e.g., pushed, hit, or was otherwise physically abusive) because of your HIV status?** *(31 participant who indicated N/A were excluded, 1 missing data)*		
No	722/733	98.5
Yes	11/733	1.5
**Have you ever been refused employment or lost a source of income or job because of your HIV status?** *(40 participant who indicated N/A were excluded, 2 missing data)*		
No	683/723	94.5
Yes	40/723	5.5
**Has your wife/husband, partner(s) or child(ren) ever experienced discrimination because of your HIV status?** *(42 participant who indicated N/A were excluded, 2 missing data)*		
No	699/721	96.9
Yes	22/721	3.1
**The impact of HIV status on various aspects of respondents’ lives**		
**My self-confidence** *(13 participant who indicated N/A were excluded, 3 missing data)*		
Has been negatively affected by my HIV status	302/749	40.3
Has been positively affected by my HIV status	118/749	15.8
Has not been affected by my HIV status	329/749	43.9
**My self-respect** *(13 participant who indicated N/A were excluded, 3 missing data)*		
Has been negatively affected by my HIV status	204/749	27.2
Has been positively affected by my HIV status	119/749	15.9
Has not been affected by my HIV status	426/749	56.9
**My ability to respect others** *(10 participant who indicated N/A were excluded, 5 missing data)*		
Has been negatively affected by my HIV status	58/750	7.7
Has been positively affected by my HIV status	158/750	21.1
Has not been affected by my HIV status	534/750	71.2
**My ability to cope with stress** *(11 participant who indicated N/A were excluded, 7 missing data)*		
Has been negatively affected by my HIV status	200/747	26.8
Has been positively affected by my HIV status	235/747	31.5
Has not been affected by my HIV status	312/747	41.7
**My ability to have close and secure relationships with others** *(12 participant who indicated N/A were excluded, 7 missing data)*		
Has been negatively affected by my HIV status	148/746	19.8
Has been positively affected by my HIV status	225/746	30.2
Has not been affected by my HIV status	373/746	50.0
**My ability to find love** *(22 participant who indicated N/A were excluded, 2 missing data)*		
Has been negatively affected by my HIV status	161/741	21.7
Has been positively affected by my HIV status	113/741	15.3
Has not been affected by my HIV status	467/741	63.0
**My desire to have children** *(38 participant who indicated N/A were excluded, 4 missing data)*		
Has been negatively affected by my HIV status	177/723	24.5
Has been positively affected by my HIV status	91/723	12.6
Has not been affected by my HIV status	455/723	62.9
**My ability to achieve personal and/or professional goals** *(17 participant who indicated N/A were excluded, 7 missing data)*		
Has been negatively affected by my HIV status	158/741	21.3
Has been positively affected by my HIV status	118/741	15.9
Has not been affected by my HIV status	465/741	62.8
**Behavioral reactions to stigma**		
**I have chosen not to attend social gatherings because of my HIV status** *(21 participant who indicated N/A were excluded, 0 missing data)*		
No	706/744	94.9
Yes	38/744	5.1
**I avoided going to a clinic or hospital when I needed to, because of my HIV status** *(20 participant who indicated N/A were excluded, 0 missing data)*		
No	647/745	86.9
Yes	98/745	13.1
**I have chosen not to apply for a job(s) because of my HIV status** *(24 participant who indicated N/A were excluded, 0 missing data)*		
No	687/741	92.7
Yes	54/741	7.3
**I have chosen not to seek social support because of my HIV status** *(28 participant who indicated N/A were excluded, 0 missing data)*		
No	660/737	89.5
Yes	77/737	10.5
**I have isolated myself from family and/or friends because of my HIV status** *(21 participant who indicated N/A were excluded, 0 missing data)*		
No	706/744	94.9
Yes	38/744	5.1
**I decided not to have sex because of my HIV status** *(27 participant who indicated N/A were excluded, 0 missing data)*		
No	674/738	91.3
Yes	64/738	8.7
**Internalized stigma**		
**It is difficult to tell people that I am HIV positive** *(1 missing data)*		
No	66/764	8.6
Yes	698/764	91.4
**Being HIV positive makes me feel dirty** *(1 missing data)*		
No	669/764	87.6
Yes	95/764	12.4
**I feel guilty that I am HIV positive** *(1 missing data)*		
No	458/764	59.9
Yes	306/764	40.1
**I am ashamed that I am HIV positive** *(3 missing data)*		
No	487/762	63.9
Yes	275/762	36.1
**I sometimes feel worthless because I am HIV positive** *(2 missing data)*		
No	546/763	71.6
Yes	217/763	28.4
**I hide my HIV status from others** *(1 missing data)*		
No	100/764	13.1
Yes	664/764	86.9
**HIV testing and treatment experience and interaction with healthcare system**		
**HIV testing choice** *(12 missing data)*		
Tested with respondent’s choice	505/753	67.1
Tested without respondent’s choice	248/753	32.9
**Reasons for testing** *(out of 505 who tested with respondent’s choice, 40 missing data)*		
A provider recommended it, or as part of other health care	136/465	29.3
I believed I was at risk for HIV	111/465	23.8
I felt sick and I/someone close to me thought it might be HIV related	133/465	28.6
As part of or because of a community-based program	40/465	8.6
It was mandatory	4/465	0.9
I just wanted to know	41/465	8.8
**Did fears about how other people would respond if you tested positive make you hesitate to get tested for HIV?** *(out of 505 who tested with respondent’s choice, 6 missing data)*		
No	318/499	63.7
Yes	181/499	36.3
**Are you currently or have you ever been on HIV treatment?** *(1 missing data)*		
No	34/764	4.5
Yes	730/764	95.5
**Did fears about someone learning your HIV status lead you to miss a dose of your HIV treatment?** *(out of 730 who were currently on HIV treatment, 9 missing data)*		
No	576/721	79.9
Yes	145/721	20.1
**Have you ever interrupted or stopped your HIV treatment?** *(out of 730 who were currently on HIV treatment, 0 missing data)*		
No	493/730	67.4
Yes	168/730	23.0
I don’t know/can’t remember	70/730	9.6
**In general, how would you describe your health at the moment?** *(3 missing data)*		
Poor	34/762	4.5
Good	399/762	52.4
Fair	329/762	43.2
**In the past 12 months, have you sought healthcare for non-HIV related health needs?** *(3 missing data)*		
No	486/762	63.8
Yes	276/762	36.2
**When you go outside the HIV clinic for general (non-HIV related) health services, do you usually disclose that you are living with HIV?**		
No	550/765	71.9
Yes	215/765	28.1
**Do you think your medical records relating to your HIV status are kept confidential?** *(2 missing data)*		
It is clear to me that my medical records are not being kept confidential	35/763	4.6
I am sure that my medical records will be kept confidential and will not be shared without my written informed consent	458/763	60.0
I don’t know if my medical records are kept confidential	270/763	35.4

A significant portion (40.5%, n = 269) of respondents indicated that they refrain from disclosing their HIV status to their spouse or sexual partner. Apart from intimate partners, voluntary disclosure was notably more prevalent among individuals in close social circles, such as family members (n = 362; 54.5%) and friends (n = 257; 38.5%), compared to those in less close settings, including neighbors (n = 43; 6.3%), employers (n = 27; 4.0%), and co-workers (n = 34; 5.1%). The experiences surrounding disclosure varied among participants. A considerable proportion of respondents found disclosing their status to close contacts to be a positive experience (n = 325; 44.0%) and reported feeling supported by them (n = 335; 45.8%). On the other hand, revealing their status to less familiar individuals was a positive experience only for 11.5% (n = 76) of participants and 15.6% (n = 105) mentioned that they felt support from them during this process. Moreover, more than half of respondents (n = 416; 56.1%) expressed that disclosing their HIV status did not become easier over time.

Overall, participants reported that experiences of enacted stigma (encounters of stigma and discrimination, including exclusion from social gatherings, religious activities or family activities, awareness of discriminatory remarks or gossip within families and social circles, verbal and physical harassment, etc.) were infrequent. The most commonly reported experience of enacted stigma was awareness of discriminatory remarks or gossip by people outside of their family due to their HIV status, with 14% (n = 102) of participants indicating they had encountered this.

The negative impact of HIV status on respondents’ lives varied across different domains. A notable proportion of participants reported experiencing adverse effects: 40.3% (n = 302) noted a negative impact on their self-confidence, followed by 27.2% (n = 204) reporting a decrease in self-respect due to their HIV status. Furthermore, 26.8% (n = 200) felt a negative impact on their ability to cope with stress, while 24.5% (n = 177) reported that their desire to have children was negatively affected. In terms of interpersonal relationships, 21.7% (n = 161) felt that their ability to find love was compromised, and 19.8% (n = 148) reported a negative impact on their ability to form close and secure relationships. Lastly, 21.3% (n = 158) noted a negative effect on their ability to achieve personal and/or professional goals.

Participants reported a range of behavioral reactions to stigma, with between 5.1% and 13.1% indicating that they had altered their behavior due to their HIV status. The highest prevalence was observed for actions such as avoiding visits to clinics or hospitals when needed (13.1%; n = 98) or refraining from seeking social support (10.5%; n = 77) due to their HIV status.

Indicators of internalized stigma were prevalent among the study participants. Many respondents reported feelings of guilt (n = 306; 40.1%), shame (n = 275; 36.1%), and worthlessness (n = 217; 28.4%) due to their HIV-positive status, while 12.4% (n = 95) expressed feeling ‘dirty’ as a result of being HIV positive. Additionally, a significant number of participants struggled with disclosing their status—over 90% (n = 698) reported difficulty in doing so, and 86.9% (n = 664) admitted to hiding their HIV status from others.

The majority of participants (n = 505; 67.1%) reported undergoing HIV testing based on their own choice. Healthcare provider recommendations (n = 136; 29.3%), symptom recognition (n = 133; 28.6%) and perceived risk assessment (111; 23.8%) were among the main reasons for taking an HIV test. Additionally, 36.3% (n = 181) admitted that fears about others’ reactions to a positive result made them hesitant to get tested. A small portion of respondents reported getting tested after their sexual partner was diagnosed with HIV (n = 35; 6.8%). A significant proportion of respondents (n = 730; 95.5%) reported currently or ever being on HIV treatment, yet 23.0% (n = 168) reported ever interrupting or stopping treatment, with 20.1% (n = 145) acknowledging missing doses due to fears that someone would know their status. Out of those who have ever interrupted or stopped HIV treatment 35.3% (n = 53) stated that they do not disclose status to their sexual partner. In the past 12 months, 36.2% (n = 276) of respondents sought healthcare for non-HIV related health needs, yet when seeking general health services outside the HIV clinic, the majority (71.9%; n = 550) did not disclose their HIV status there.

### Factors associated with HIV status disclosure

In the bivariate analysis (Table 2), several sociodemographic factors showed significant associations with the disclosure of HIV status to sexual partner. Individuals aged 30 and above were more likely to disclose their HIV status compared to those aged 18–29, with increasing odds ratios observed with older age groups. HIV status knowledge for 2–5 years, 6–10 years, and more than 10 years was also significantly associated with status disclosure. Knowing a partner’s HIV status was associated with status disclosure to sexual partner: Individuals who knew their partner was HIV positive were more likely to disclose their own status compared to those who knew their partner was HIV negative. Conversely, those who were unsure about their partner’s HIV status were less likely to disclose their own status than individuals whose partners were HIV negative. Belonging to the group of refugees or asylum seekers and having a history of incarceration were both associated with HIV status disclosure. Individuals who identified as refugees or asylum seekers were more likely to disclose their status compared to those who did not belong to this group, and those with a history of incarceration were also more likely to disclose their HIV status to their sexual partner compared to those without such experience. In addition, individuals who were part of a PLHIV network or support group had an increased odds of status disclosure to their sexual partner compared to those who were not part of such a group.

**Table 2:** Sociodemographic characteristics, treatment experience and interaction with healthcare system by status disclosure to sexual partner.

Variable	Do not disclose HIV status to sexual partner n (%)	Disclose HIV status to sexual partner	P value*	OR** (95% CI)
**Sociodemographic**				
**Sex at birth**			0.284	
Male	185 (41.8)	258 (58.2)		Ref
Female	80 (37.4)	134 (62.6)		1.20 (0.86–1.68)
**Age group**			**0.001**	
18-29	62 (56.4)	48 (43.6)		Ref
30-39	92 (42.4)	125 (57.6)		**1.75 (1.10–2.79)**
40-49	64 (34.0)	124 (66.0)		**2.50 (1.54–4.06)**
50+	51 (34.0)	99 (66.0)		**2.51 (1.51–4.16)**
**Length of time knowing HIV status**			**0.004**	
Less than 2 years	20 (62.5)	12 (37.5)		Ref
2-5 years	93 (39.7)	141 (60.3)		**2.53 (1.18–5.41)**
6-10 years	78 (39.2)	121 (60.8)		**2.59 (1.20–5.59)**
More than 10 years	64 (35.8)	115 (64.2)		**2.99 (1.38–6.52)**
Do not remember	14 (70.0)	6 (30.0)		0.71 (0.22–2.36)
**Partner’s HIV status**			**< 0.001**	
Negative	45 (22.1)	159 (77.9)		Ref
Positive	26 (13.4)	168 (86.6)		**1.83 (1.10–3.10)**
Unsure about the HIV status of partner	15 (46.9)	17 (53.1)		**0.32 (0.15–0.69)**
**Education level**			0.187	
Lower level education	179 (42.0)	247 (58.0)		Ref
Higher level education	86 (36.7)	148 (63.3)		1.25 (0.89–1.73)
**Current work status**			0.903	
Unemployed	104 (40.6)	152 (59.4)		Ref
Having some form of employment	163 (40.2)	243 (59.8)		1.02 (0.74–1.40)
**How often have you been unable to meet basic needs**			0.414	
Never	51 (35.4)	93 (64.6)		Ref
Some of the time	169 (41.7)	236 (58.3)		0.77 (0.52–1.14)
Most of the time	44 (40.4)	65 (59.6)		0.81 (0.46–1.35)
**Belong to a racial, ethnic, or religious minority**			0.686	
No	260 (40.9)	376 (59.1)		Ref
Yes	5 (31.3)	11 (68.7)		1.52 (0.52–4.43)
Prefer not to answer	4 (33.3)	8 (66.7)		1.38 (0.41–4.64)
**Have been a member of people living with a disability**			0.968	
No	248 (40.7)	362 (59.3)		Ref
Yes	17 (38.6)	27 (61.4)		1.09 (0.58–2.04)
Prefer not to answer	4 (40.0)	6 (60.0)		1.03 (0.29–3.68)
**Belong to refugee or asylum seeker**			**0.062**	
No	261 (41.3)	371 (58.7)		Ref
Yes	4 (17.4)	19 (82.6)		**3.34 (1.12–9.94)**
Prefer not to answer	4 (44.4)	5 (55.6)		0.88 (0.23–3.31)
**Belong to migrant workers**			0.807	
No	253 (40.4)	374 (59.6)		Ref
Yes	12 (46.2)	14 (53.8)		0.79 (0.36–1.73)
Prefer not to answer	4 (44.4)	5 (55.6)		0.85 (0.22–3.18)
**Belong to internally displaced persons**			0.100	
No	249 (40.5)	366 (59.5)		Ref
Yes	16 (40.0)	24 (60.0)		1.02 (0.53–1.96)
Prefer not to answer	4 (44.4)	5 (55.6)		0.85 (0.22–3.20)
**Have been incarcerated/in prison**			**0.024**	
No	264 (41.4)	374 (58.6)		Ref
Yes	1 (7.1)	13 (92.9)		**9.18 (1.19–70.6)**
Prefer not to answer	4 (40.0)	6 (60.0)		1.06 (0.30–3.79)
**Belong to a network or support group of PLHIV**			**0.016**	
No	242 (42.4)	329 (57.6)		Ref
Yes	25 (28.7)	62 (71.3)		**1.82 (1.11–2.99)**
**HIV testing and treatment experience and interaction with healthcare system**				
**HIV testing choice**			**0.019**	
Tested without respondent’s choice	102 (46.2)	119 (53.8)		Ref
Tested with respondent’s choice	159 (36.6)	275 (63.4)		**1.48 (1.07–2.05)**
**Reasons for testing**			**0.042**	
A provider recommended it, or as part of other health care	48 (42.1)	66 (57.9)		Ref
I believed I was at risk for HIV	28 (29.5)	67 (70.5)		1.74 (0.98–3.10)
I felt sick and I/someone close to me thought it might be HIV related	37 (32.2)	78 (67.8)		1.53 (0.89–2.63)
As part of or because of a community-based program	18 (52.9)	16 (47.1)		0.65 (0.30–1.40)
It was mandatory	1 (33.3)	2 (66.7)		1.45 (0.13–16.51)
I just wanted to know	19 (50.0)	19 (50.0)		0.73 (0.35–1.52)
**Did fears about how other people would respond if you tested positive make you hesitate to get tested for HIV?**			0.683	
No	100 (37.6)	166 (62.4)		Ref
Yes	57 (35.6)	103 (64.4)		1.09 (0.72–1.64)
**Are you currently or have you ever been on HIV treatment?**			**< 0.001**	
No	25 (78.1)	7 (21.9)		Ref
Yes	244 (38.6)	388 (61.4)		**5.68 (2.42–13.33)**
**Did fears about someone learning your HIV status lead you to miss a dose of your HIV treatment?**			0.860	
No	193 (38.4)	310 (61.6)		Ref
Yes	45 (37.5)	75 (62.5)		1.04 (0.69–1.57)
**Have you ever interrupted or stopped your HIV treatment?**			0.188	
No	164 (38.4)	263 (61.6)		Ref
Yes	53 (35.3)	97 (64.7)		0.99 (0.99–1.00)
**In general, how would you describe your health at the moment?**			**< 0.001**	
Poor	11 (34.4)	21 (65.6)		Ref
Good	175 (49.9)	176 (50.1)		0.53 (0.25–1.13)
Fair	81 (29.0)	198 (71.0)		1.28 (0.59–1.78)
**In the past 12 months, have you sought healthcare for non-HIV related health needs?**			**0.010**	
No	191 (43.8)	245 (56.2)		Ref
Yes	76 (33.5)	151 (66.5)		**1.55 (1.11–2.16)**
**When you go outside the HIV clinic for general (non-HIV related) health services, do you usually disclose that you are living with HIV?**			0.177	
No	203 (42.0)	280 (58.0)		Ref
Yes	66 (36.3)	116 (63.7)		1.27 (0.90–1.81)
**Do you think your medical records relating to your HIV status are kept confidential?**			**0.001**	
It is clear to me that my medical records are not being kept confidential	11 (34.4)	21 (65.6)		Ref
I am sure that my medical records will be kept confidential and will not be shared without my written informed consent	183 (46.1)	214 (53.9)		0.61 (0.29–1.30)
I don’t know if my medical records are kept confidential	74 (31.6)	160 (68.4)		1.13 (0.52–2.47)

*P-values were calculated using chi-squared tests. For categories with cell counts less than 5, Fisher’s exact test was used.

**Unadjusted ORs from simple logistic regression

Testing and treatment experience also showed significant associations with status disclosure. Individuals who were tested for HIV with their own choice had higher odds of disclosing their HIV status to sexual partner compared to those who were tested without their choice. Individuals on HIV treatment had more than five times the odds of disclosing their HIV status to sexual partner compared to those not on treatment.

Table 3 presents a comparative analysis of composite scores across stigma-discrimination domains and HIV status disclosure to sexual partner. Both composite scores and individual items were examined to assess associations between stigma, discrimination, and status disclosure, with individual items detailed in S1 Table. Composite scores for disclosure experiences, derived from five related questions showed a strong positive association with HIV status disclosure. Participants who disclosed their status to sexual partner reported significantly more positive experiences than those who did not disclose (P < 0.001). Composite scores assessing enacted stigma, which included nine related questions, indicated that individuals who disclosed their HIV status to sexual partner reported higher levels of enacted stigma compared to non-disclosers (P < 0.001). The composite score for internalized stigma, derived from six related questions, showed that individuals who disclosed their HIV status to sexual partner had significantly lower levels of internalized stigma than non-disclosers (P = 0.001).

**Table 3:** Comparative analysis of composite scores across stigma-discrimination domains and HIV status disclosure to sexual partner.

Domain	# of variables	Response and scores		Average Composite Score for “Do not disclose status to sexual partner”	Average Composite Score for “Disclose status to sexual partner”	T-test P-value
Disclosure experience	5	Disagree	−2	−0.4253	0.3485	**< 0.001**
		Somewhat Agree	1			
		Agree	2			
Enacted stigma	9	No	−2	−1.9203	−1.7621	**< 0.001**
		Yes but not in 12m	1			
		Yes in 12m	2			
Impact on lives	8	Negative	−1	−0.0300	−0.0245	0.874
		No Affection	0			
		Positive	1			
Behavioral reactions to stigma	6	No	−1	−0.8604	−0. 8170	0.152
		Yes	1			
Internalized stigma	6	No	−1	0.0595	−0.0773	**0.001**
		Yes	1			

The logistic regression analysis revealed several significant predictors of HIV status disclosure to sexual partner (Table 4). Individuals aged 40–49 were almost four times more likely to disclose their HIV status to a sexual partner (aOR: 3.83; 95% CI: 1.35–10.88) compared to those aged 18–29. Those aged 50 and above were more than six times more likely to disclose their status (aOR: 6.44; 95% CI: 1.96–21.23) compared to the 18–29 age group. Participants whose partners were known to be HIV-positive were more likely (aOR: 2.37; 95% CI: 1.15–4.88) to disclose their status compared to those whose partners were known to be HIV negative, while those unsure about partners’ HIV status were less likely (aOR: 0.27; 95% CI: 0.09–0.80) to disclose their status compared to those whose partners were known to be HIV negative. Finally, higher scores for disclosure experience, indicating a more positive experience (aOR: 1.97; 95% CI: 1.49–2.62), and higher scores for enacted stigma, suggesting more instances of such experience (aOR: 6.77; 95% CI: 1.66–27.63), were both positively associated with the likelihood of disclosing HIV status to sexual partner.

**Table 4:** Multivariate Regression analysis between sociodemographic predictors, experiences of stigma discrimination, experiences of interactions with healthcare and HIV status disclosure to sexual partner.

Disclosure HIV status to sexual partner				
Variable	Category	aOR	95% CI	P value
Sex at birth	Male	Ref		
	Female	1.10	0.51–2.21	0.875
Age	18-29	Ref		
	30-39	1.90	0.75–4.76	0.174
	40-49	**3.83**	**1.35–10.88**	**0.012**
	50+	**6.44**	**1.96–21.23**	**0.002**
Length of time knowing HIV status	Less than 2 years	Ref		
	2-4 years	0.84	0.12–5.80	0.858
	5-10 years	0.94	0.13–6.75	0.947
	Cannot remember	0.39	0.05–2.96	0.362
Partner’s HIV status	Negative	Ref		
	Positive	**2.37**	**1.15–4.88**	**0.019**
	Unsure about the HIV status of partner	**0.27**	**0.09–0.80**	**0.018**
Education level	Lower level	Ref		
	Higher level	1.07	0.56–2.07	0.832
Belong to refugee or asylum seeker	No	Ref		
	Yes	0.94	0.63–1.41	0.778
Have been incarcerated/in prison	No	Ref		
	Yes	1.05	0.71–1.56	0.809
Belong to a network or support group of PLHIV	No	Ref		
	Yes	1.25	0.41–3.83	0.699
HIV testing choice	Tested without respondent’s choice	Ref		
	Tested with respondent’s choice	0.73	0.35–1.51	0.390
Are you currently or have you ever been on HIV treatment?	No	Ref		
	Yes	0.41	0.04–4.39	0.461
In the past 12 months, have you sought healthcare for non-HIV related health needs?	No	Ref		
	Yes	2.09	1.01–4.32	0.047
Stigma-discrimination composite scores	Disclosure experience score	**1.97**	**1.49–2.62**	**< 0.001**
	Enacted stigma score	**6.77**	**1.66–27.63**	**0.008**
	Impact on lives score	0.47	0.21–1.07	0.073
	Behavioral reactions to stigma	1.26	0.39–4.03	0.700
	Internalized stigma score	0.52	0.25–1.10	0.089

## Discussion

This study provides insight into the sociodemographic, psychosocial, and experiential factors associated with HIV status disclosure to sexual partner among PLHIV in the country of Georgia. The findings indicate that a considerable proportion of respondents (23.0%) reported ever interrupting or stopping HIV treatment, with 20.1% acknowledging that fear of disclosure contributed to missing doses. Among those with treatment interruptions, 35.3% reported that they do not disclose their status to their sexual partner. This is particularly concerning given the implications for viral suppression and potential for increased HIV transmission [26], highlighting the critical role of status disclosure to sexual partner. Moreover, the high levels of internalized stigma and reluctance to disclose HIV status, even in healthcare settings, remain a significant concern. Notably, 71.9% of participants did not disclose their status while receiving general health services, highlighting the ongoing challenges in ensuring comprehensive care and support for PLHIV. These findings underline the importance of targeted interventions to facilitate disclosure, particularly in populations where treatment interruption poses a significant risk to both individual health and public health goals, including the prevention of HIV transmission.

Our results highlight significant sociodemographic association with HIV status disclosure, with older individuals (over 40) more likely to disclose to sexual partner than younger ones. This finding aligns with some existing literature, including those from EECA region [27–29]. Greater life experience and the adaptability that often accompanies older age may enhance the ability to navigate complex situations, thereby facilitating HIV status disclosure. However, other studies have reported opposite results, with younger PLHIV being more likely to disclose their status. Similar findings have been observed not only among the general PLHIV population [30] but also within specific groups, including MSM [31] and pregnant PLHIV [32]. This suggests that age-related disclosure behaviors are complex and may be influenced by a range of contextual factors that warrant further investigation.

In our study, participants whose partners were known to be HIV-positive were more likely to disclose their status, which may be due to a perception of shared experience and mutual understanding. On the other hand, participants who were unsure about partner’s HIV status were less likely to disclose their own status. These findings are particularly concerning in light of the rates of treatment interruption and missed doses due to fear of disclosure reported by the study participants. Partner’s HIV-positive status as a predictor of disclosure has also been observed in a study from the Russian Federation [33], while a study from Ukraine [27] reported that those who did not know their partner’s HIV status had higher odds of non-disclosure.

HIV status disclosure to sexual partner remains a complex issue, with fear of stigma and discrimination and negative outcomes acting as a significant barrier [34–38]. Our multivariate logistic regression analysis revealed that higher scores for positive disclosure experiences were strongly associated with an increased likelihood of HIV status disclosure to sexual partner. Consistent with Chaudoir and Fisher’s Disclosure Process Model [21], this suggests that past positive experiences may create an expectation of future positive outcomes, encouraging individuals to disclose their status more readily, as they anticipate supportive reactions from their partners. Conversely, perceived stigma has been shown to be negatively associated with HIV disclosure [39–41], highlighting the crucial role that both past experiences and perceived social support play in influencing disclosure behaviors. Additionally, we found that disclosure of HIV status to a sexual partner was significantly associated with experiences of enacted stigma, including social exclusion, discriminatory remarks, and harassment. Given the cross-sectional design of our research, we cannot ascertain the causal direction of this association: whether experiencing enacted stigma motivates individuals to disclose their status more frequently, or if disclosure itself leads to increased experiences of enacted stigma. The latter has been documented in previous studies from the EECA on the consequences of HIV disclosure [42–44]. Given the potential negative costs of disclosure, it is essential to provide personalized decision support to PLHIV in making informed decisions about disclosure on an individual basis.

Our study is not without limitations. The PLHIV Stigma Index 2.0 has been widely used globally but no formal psychometric validation studies are available, especially in individual country contexts. While formal validation studies of the PLHIV Stigma Index 2.0 tool are limited, the use of the tool across multiple countries in the region provides indirect support for its external validity. Notably, one key indicator—avoidance of healthcare by PLHIV due to stigma, which is also used in the sample size calculator for the Stigma Index—has been measured in Ukraine (12%) [45], Georgia (13.2%) [46], and Kazakhstan (10.2%) [47] using comparable methodology. These countries share similarities in the organization of HIV care, including public sector-led vertical service delivery and post-Soviet health system structures. The consistency of findings across these settings suggests that the tool captures relevant constructs. The cross-sectional design restricts our ability to establish causal relationships between the variables and HIV status disclosure to sexual partner. The outcome variable, which only measures whether participants disclosed their HIV status to their partners, lacks differentiation between regular and casual partners, potentially obscuring variations in disclosure behaviors across different types of relationships. Additionally, the reliance on self-reported data introduces the potential for social desirability bias, where participants may underreport or over report certain behaviors or experiences. These inherent limitations should be considered when interpreting the results and their implications. Despite these limitations, the study provides valuable insights into the factors associated with HIV status disclosure to sexual partner among Georgian PLHIV.

## Conclusion

This study, based on the first large-scale investigation of stigma among Georgian PLHIV, provides critical insights into the factors associated with HIV status disclosure to sexual partner. Our findings reveal that older age, knowing partner’s HIV status, positive disclosure experiences and enacted stigma were positively associated with disclosing status to sexual partner. Considering the frequency of treatment interruptions among Georgian PLHIV, it is crucial to develop targeted interventions that support individuals in disclosing their status to sexual partner, ultimately aiming to prevent HIV transmission. These findings underscore the importance of developing tailored strategies to address the diverse factors that influence HIV status disclosure and support PLHIV in disclosure decision-making process, ultimately contributing to improved health outcomes and more effective HIV prevention efforts in Georgia and EECA region. Future research should continue to explore these dynamics, particularly focusing on the role of stigma and its interplay with other psychosocial factors, to inform the design of more effective interventions aiming to enhance health outcomes and prevention efforts for PLHIV in the region.

## Supporting information

S1 Table Comparison of individual question responses for disclosure experiences, enacted stigma, the impact of HIV status on various aspects of respondents’ lives, behavioral reactions to stigma and internalized stigma by status disclosure to sexual partner. (DOCX)
