# Supplementary material for: Disclosing HIV status to sexual partner: Findings from a People Living with HIV Stigma Index 2.0 study in the country Georgia
Source: PLoS One. 2025 Oct 8;20(10):e0331919. doi: 10.1371/journal.pone.0331919 (PMC12507297; doi:10.1371/journal.pone.0331919)
Supplement: S1 Table — (DOCX) [file pone.0331919.s001.docx]

**Additional file 1.** Comparison of individual question responses for disclosure experiences, enacted stigma, the impact of HIV status on various aspects of respondents’ lives, behavioral reactions to stigma and internalized stigma by status disclosure to sexual partner.

| **Variable** | **Do not disclose HIV status to sexual partner**  **n (%)** | **Disclose HIV status to sexual partner**  **n (%)** | **P value*** | **OR****  **(95% CI)** |
| --- | --- | --- | --- | --- |
| **Disclosure experiences** | | | | |
| **Disclosing your HIV status to people you are close to has been a positive experience.** |  |  | **<0.001** |  |
| Disagree | 91 (68.4) | 42 (31.6) |  | Ref |
| Agree | 74 (25.7) | 214 (74.3) |  | **6.27 (3.99-9.84)** |
| Somewhat agree | 89 (40.1) | 133 (59.9) |  | **3.24 (2.06-5.09)** |
| **People you are close to were supportive when they first learned about your HIV status.** |  |  | **<0.001** |  |
| Disagree | 96 (65.3) | 51 (34.7) |  | Ref |
| Agree | 76 (26.4) | 212 (73.6) |  | **5.25 (3.42-8.06)** |
| Somewhat agree | 81 (40.1) | 121 (59.9) |  | **2.81 (1.81-4.37)** |
| **Disclosing your HIV status to people you don’t know very well has been a positive experience.** |  |  | **<0.001** |  |
| Disagree | 166 (47.4) | 184 (52.6) |  | Ref |
| Agree | 21 (32.8) | 43 (67.2) |  | **1.85 (1.05-3.24)** |
| Somewhat agree | 50 (30.1) | 116 (69.9) |  | **2.09 (1.41-3.09)** |
| **People you don’t know very well were supportive when they first learned about your HIV status.** |  |  | **0.001** |  |
| Disagree | 157 (47.6) | 173 (52.4) |  | Ref |
| Agree | 29 (31.9) | 62 (68.1) |  | **1.94 (1.18-3.17)** |
| Somewhat agree | 55 (32.4) | 115 (67.6) |  | **1.89 (1.29-2.79)** |
| **Disclosing your HIV status has become easier over time.** |  |  | **<0.001** |  |
| Disagree | 177 (49.4) | 181 (50,6) |  | Ref |
| Agree | 31 (24.0) | 98 (76.0) |  | **3.09 (1.96-4.86)** |
| Somewhat agree | 48 (30.6) | 109 (69.4) |  | **2.22 (1.49-3.30)** |
| **Enacted Stigma** | | | | |
| **Have you ever been excluded from social gatherings or activities because of your HIV status?** |  |  | **0.009** |  |
| No | 256 (41.8) | 357 (58.2) |  | Ref |
| Yes | 2 (11.1) | 16 (88.9) |  | **5.74 (1.31-25.16)** |
| **Have you ever been excluded from religious activities or places of worship because of your HIV status?** |  |  | 0.190 |  |
| No | 253 (41.1) | 362 (58.9) |  | Ref |
| Yes | 3 (23.1) | 10 (76.9) |  | 2.32 (0.63-8.54) |
| **Have you ever been excluded from family activities because of your HIV status?** |  |  | **0.003** |  |
| No | 255 (41.7) | 356 (58.3) |  | Ref |
| Yes | 2 (9.5) | 19 (90.5) |  | **6.80 (1.57-29.47)** |
| **Have you ever been aware of family members making discriminatory remarks or gossiping about you because of your HIV status?** |  |  | **0.001** |  |
| No | 245 (42.8) | 327 (57.2) |  | Ref |
| Yes | 12 (20.3) | 47 (79.7) |  | **2.93 (1.52-5.65)** |
| **Have you ever been aware of other people (other than family members) making discriminatory remarks or gossiping about you because of your HIV status?** |  |  | **0.001** |  |
| No | 237 (43.4) | 309 (56.6) |  | Ref |
| Yes | 22 (25.3) | 65 (74.7) |  | **2.26 (1.35-3.78)** |
| **Has someone ever verbally harassed you (e.g., yelled, scolded, or was otherwise verbally abusive) because of your HIV status?** |  |  | **0.015** |  |
| No | 252 (42.1) | 347 (57.9) |  | Ref |
| Yes | 10 (23.3) | 33 (76.7) |  | **2.39 (1.15-4.95)** |
| **Has someone every physically harassed or hurt you (e.g., pushed, hit, or was otherwise physically abusive) because of your HIV status?** |  |  | 0.045 |  |
| No | 261 (41.5) | 368 (58.5) |  | Ref |
| Yes | 1 (10.0) | 9 (90.0) |  | 6.38 (0.80-50.68) |
| **Have you ever been refused employment or lost a source of income or job because of your HIV status?** |  |  | **<0.001** |  |
| No | 252 (42.1) | 347 (57.9) |  | Ref |
| Yes | 3 (9.1) | 30 (90.9) |  | **7.26 (2.19-24.06)** |
| **Has your wife/husband, partner(s) or child(ren) ever experienced discrimination because of your HIV status?** |  |  | **0.009** |  |
| No | 256 (41.7) | 358 (58.3) |  | Ref |
| Yes | 2 (11.1) | 16 (88.9) |  | **5.72 (1.30-25.09)** |
| **The impact of HIV status on various aspects of respondents’ lives** | | | | |
| **My self-confidence** |  |  | **<0.001** |  |
| Has been negatively affected by my HIV status | 76 (29.2) | 184 (70.8) |  | Ref |
| Has been positively affected by my HIV status | 34 (32.4) | 71 (67.6) |  | 0.86 (0.52-1.40) |
| Has not been affected by my HIV status | 152 (52.9) | 135 (47.1) |  | **0.36 (0.25-0.52)** |
| **My self-respect** |  |  | **<0.001** |  |
| Has been negatively affected by my HIV status | 57 (32.4) | 119 (57.6) |  | Ref |
| Has been positively affected by my HIV status | 32 (30.2) | 74 (69.8) |  | 1.10 (0.65-1.86) |
| Has not been affected by my HIV status | 172 (46.6) | 197 (53.4) |  | **0.54 (0.37-0.79)** |
| **My ability to respect others** |  |  | **0.046** |  |
| Has been negatively affected by my HIV status | 15 (30.0) | 35 (70.0) |  | Ref |
| Has been positively affected by my HIV status | 48 (34.3) | 92 (65.7) |  | 0.82 (0.40-1.65) |
| Has not been affected by my HIV status | 201 (43.4) | 262 (56.6) |  | 0.55 (0.29-1.05) |
| **My ability to cope with stress** |  |  | **<0.001** |  |
| Has been negatively affected by my HIV status | 49 (29.5) | 117 (70.5) |  | Ref |
| Has been positively affected by my HIV status | 63 (29.7) | 149 (70.3) |  | 0.99 (0.63-1.54) |
| Has not been affected by my HIV status | 150 (55.1) | 122 (44.9) |  | **0.34 (0.22-0.51)** |
| **My ability to have close and secure relationships with others** |  |  | **<0.001** |  |
| Has been negatively affected by my HIV status | 30 (24.2) | 94 (75.8) |  | Ref |
| Has been positively affected by my HIV status | 65 (32.7) | 134 (67.3) |  | 0.65 (0.39-1.09) |
| Has not been affected by my HIV status | 167 (50.9) | 161 (49.1) |  | **0.30 (0.19-0.48)** |
| **My ability to find love** |  |  | 0.065 |  |
| Has been negatively affected by my HIV status | 59 (43.4) | 77 (56.6) |  | Ref |
| Has been positively affected by my HIV status | 30 (29.7) | 71 (70.3) |  | **1.81 (1.05-3.12)** |
| Has not been affected by my HIV status | 170 (41.5) | 240 (58.5) |  | 1.08 (0.73-1.60) |
| **My desire to have children** |  |  | **0.001** |  |
| Has been negatively affected by my HIV status | 54 (36.7) | 93 (63.3) |  | Ref |
| Has been positively affected by my HIV status | 19 (23.2) | 63 (76.8) |  | **1.92 (1.04-3.55)** |
| Has not been affected by my HIV status | 181 (45.0) | 221 (55.0) |  | 0.70 (0.48-1.04) |
| **My ability to achieve personal and/or professional goals** |  |  | **<0.001** |  |
| Has been negatively affected by my HIV status | 42 (31.1) | 93 (68.9) |  | Ref |
| Has been positively affected by my HIV status | 31 (29.0) | 76 (71.0) |  | 1.10 (0.63-1.92) |
| Has not been affected by my HIV status | 184 (45.7) | 219 (54.3) |  | **0.53 (0.35-0.81)** |
| **Behavioral reactions to stigma** | | | | |
| **I have chosen not to attend social gatherings** |  |  | 0.286 |  |
| No | 255 (41.6) | 358 (58.4) |  | Ref |
| Yes | 11 (32.4) | 23 (67.6) |  | 1.48 (0.71-3.10) |
| **I avoided going to a clinic or hospital when I needed to** |  |  | **0.020** |  |
| No | 241 (42.7) | 323 (57.3) |  | Ref |
| Yes | 25 (29.4) | 60 (70.6) |  | **1.79 (1.09-2.93)** |
| **I have chosen not to apply for a job(s)** |  |  | 0.048 |  |
| No | 253 (42.4) | 344 (57.6) |  | Ref |
| Yes | 13 (27.7) | 34 (72.3) |  | 1.92 (0.90-3.71) |
| **I have chosen not to seek social support** |  |  | 0.496 |  |
| No | 239 (41.6) | 335 (58.4) |  | Ref |
| Yes | 25 (37.3) | 42 (62.7) |  | 1.19 (0.71-2.02) |
| **I have isolated myself from family and/or friends** |  |  | 0.569 |  |
| No | 254 (41.4) | 360 (58.6) |  | Ref |
| Yes | 12 (36.4) | 21 (63.6) |  | 1.23 (0.59-2.55) |
| **I decided not to have sex** |  |  | 0.352 |  |
| No | 240 (40.6) | 351 (59.4) |  | Ref |
| Yes | 25 (47.2) | 28 (52.8) |  | 0.76 (0.43-1.34) |
| **Internalized stigma** | | | | |
| **It is difficult to tell people that I am HIV positive** |  |  | 0.106 |  |
| No | 17 (30.4) | 39 (69.6) |  | Ref |
| Yes | 252 (41.5) | 356 (58.6) |  | 0.61 (0.34-1.11) |
| **Being HIV positive makes me feel dirty** |  |  | **0.035** |  |
| No | 227 (39.0) | 355 (61.0) |  | Ref |
| Yes | 42 (51.2) | 40 (48.8) |  | **0.60 (0.38-0.96)** |
| **I feel guilty that I am HIV positive** |  |  | 0.838 |  |
| No | 162 (40.2) | 241 (59.8) |  | Ref |
| Yes | 107 (41.0) | 154 (59.0) |  | 0.96 (0.70-1.32) |
| **I am ashamed that I am HIV positive** |  |  | **0.002** |  |
| No | 153 (36.1) | 271 (63.9) |  | Ref |
| Yes | 116 (48.5) | 123 (51.5) |  | **0.59 (0.43-0.82)** |
| **I sometimes feel worthless because I am HIV positive** |  |  | **<0.001** |  |
| No | 174 (36.0) | 309 (64.0) |  | Ref |
| Yes | 95 (52.8) | 85 (47.2) |  | **0.50 (0.35-0.71)** |
| **I hide my HIV status from others** |  |  | **0.037** |  |
| No | 26 (30.2) | 60 (69.8) |  | Ref |
| Yes | 243 (42.0) | 335 (58.0) |  | **0.59 (0.36-0.97)** |
| *P-values were calculated using chi-squared tests. For categories with cell counts less than 5, Fisher's exact test was used.  **Unadjusted ORs from simple logistic regression | | | | |
